# Psychometric Properties of the Screen for Child Anxiety Related Emotional Disorders (SCARED) in a Non-Clinical Sample of Children and Adolescents in Saudi Arabia

**DOI:** 10.1007/s10578-015-0589-0

**Published:** 2015-09-30

**Authors:** Arwa Arab, Mogeda El Keshky, Julie A. Hadwin

**Affiliations:** Department of Psychology, Faculty of Arts and Humanities, King Abdulaziz University, Jeddah, Saudi Arabia; Developmental Brain-Behaviour Laboratory, Psychology Academic Unit, University of Southampton, Highfield, Southampton, SO17 1BJ UK

**Keywords:** Screen for Child Anxiety Disorder (SCARED), Psychometric properties, Model fit, Children, Adolescents, Saudi Arabia

## Abstract

This paper examined the reliability, convergent validity and factor structure of the self-report Screen for Child Anxiety Disorders (SCARED; Birmaher et al. in J Am Acad Child Adolesc Psychiatry 36:545–553, 1997) in a large community sample of children and adolescents in Saudi Arabia. The questionnaire showed moderate to high internal consistency and satisfactory test–retest reliability over a 2 week period. In addition, there were significant positive correlations between reported anxiety symptoms with parent report behavioural difficulties. The five factor structure model of the SCARED also had a good model fit in this population. The results showed that self-report anxiety symptoms decreased with age (for boys and not girls) and were higher in adolescent girls. The results suggest that the SCARED could be useful in this population to identify individuals who are at risk of developing anxiety disorders in childhood with a view to implementing prevention and intervention methods to ensure positive developmental outcome over time.

## Introduction

The Diagnostic and Statistical Manual for Mental Disorders (DSM-5; [5]) identifies several anxiety disorders that can develop in childhood or adolescence (e.g., separation anxiety disorder, specific phobia and social anxiety disorder). Anxiety can have a significant negative impact on daily life for young people and is associated with low quality peer relationships [18], lowered attendance in school [32, 41, 46] and academic underachievement [26, 40]. Moreover, it can follow a chronic pathway from childhood through to adulthood, placing children and adolescents at risk for further mental and physical health difficulties [18, 42]. Researchers have highlighted that anxiety symptoms can be less visible in development compared with other disorders (review by [17]); emphasizing the importance of self-report measurements that are reliable and valid [20, 25].

One widely used DSM based anxiety questionnaire for children and adolescents aged 8–18 years is the Screen for Child Anxiety Disorders (SCARED; [7]). The 41 items in this measure were developed in English and based on anxiety symptoms in clinical populations. The questionnaire measures total anxiety and includes five factors or subscales (separation anxiety disorder, generalized anxiety, social phobia (now social anxiety disorder), school phobia and panic/somatic symptoms). In addition, it provides a cut-off to identify young people who are experiencing clinically significant symptom levels. Several papers have reported good reliability (e.g., test–re-test and internal consistency; [6, 7, 9, 20, 31, 43]). In addition, the questionnaire has been found to have good convergent [9, 20, 36, 39, 43] and discriminant validity [6, 7, 36, 43]. Since its development, the SCARED has been translated into several languages and has used as an anxiety screening tool in Europe, (e.g. [16, 19, 29]) and worldwide (e.g. [11, 33]). In addition, several studies have found support for its five factor structure (e.g. [9, 19, 28, 31, 38, 43]).

In Western cultures, anxiety represents one of the most prevalent disorders in development, with up to 15 % of children and adolescents meeting the diagnostic criteria [45]. Studies typically report that while negative affect is similar in boys and girls in childhood [8, 21, 42], gender differences start to emerge in adolescence; with females showing elevated and more stable symptoms compared with males [8, 30]. Few studies have, however, explored the prevalence of anxiety in Arab countries in child and adolescent populations. In one recent study, Al-Sughayr and Ferwana [2] measured mental health difficulties in adolescents and young adults in Saudi Arabia (including somatic symptoms, anxiety, depression and social dysfunction). This paper highlighted that 48 % of individuals reported symptoms consistent with a psychiatric disorder and that symptom severity was higher in females compared with males. Other studies have found similar levels (49 %) of anxiety in adolescent boys in Saudi Arabia [1]. A review paper considering anxiety disorders in adults in Saudi Arabia also highlighted gender differences, although prevalence rates in some studies were lower (from 7 to 33 %; [44]). Consideration of previous research highlights a need to understand more clearly child and adolescent anxiety symptoms in Arabic cultures to develop a better understanding of prevalence rates across development and between gender and with a view to addressing causal factors and prevention and intervention strategies in the longer term.

Previous studies generally support the use of SCARED as a reliable and valid screening tool to measure anxiety symptoms. Only one study to-date has explored the psychometric properties of SCARED in an Arabic speaking population. Hariz et al. [31] demonstrated satisfactory psychometric properties (related to internal consistency and good discriminant, convergent and divergent validity) for total anxiety scores in a clinical population aged 9–17 years in Lebanon. The aim of the current study was to extend this research to explore the psychometric properties of this questionnaire in a community sample of children and adolescents in Saudi Arabia. It measured anxiety symptoms in two age groups (pre-adolescent and adolescent) and between gender. Following previous research (e.g. [42]), it was anticipated that anxiety symptoms would increase with age and that gender differences would be most evident in adolescence. In addition, the study measured the reliability (internal consistency and test–re-test reliability) and convergent validity of the SCARED. Following Hariz et al. [31], it considered the association between self-report anxiety symptoms with a parent measure of emotional symptoms and behavioural difficulties. It also extended previous research to explore the factor structure of this measure in an Arabic population. The study has implications for understanding symptoms of anxiety in a developing population in Saudi Arabia and the use of the SCARED as an effective screening tool in this culture.

## Methods

### Participants

Children and adolescents living in Jeddah in the west of Saudi Arabia (n = 1100) aged between 9 and 15 years were randomly selected to take part from nine basic and elementary schools. All schools were from metropolitan regions, had single gender populations and represented diverse socio-economic backgrounds (as measured using postcodes). Of the initial 1100 students, 161 were excluded due to incomplete questionnaires. The final sample consisted of 939 participants (mean age = 11.70 years, *SD* = 1.82, 562 females); 609 children from basic (mean age = 10.59, *SD* = 1.14 years old, range = 9–12 years old) and 330 adolescents from elementary schools (mean age = 13.76 years, *SD* = .74 years old, range = 13–15 years old). Participation was dependent on gaining appropriate permissions from the School Boards and parents. Students completed the SCARED during regular classroom hours. In addition, a subgroup of children and adolescents (n = 223, mean age = 11.76, SD = 1.84) completed the SCARED again 2 weeks later to measure test–re-test reliability. In order to ensure data collection was administered in the same way between schools, research assistants read and followed manual guidelines.

### Measures

#### The Screen for Child Anxiety Related Disorders (SCARED)

We used the 41 item child self-report version of the SCARED to measure anxiety symptoms in children and adolescents [6, 7]. Thirteen items relate to panic/somatic symptoms (PN), 8 each to generalized anxiety (GD) and separation anxiety (SP), 7 to social phobia (SOC) and 4 to school phobia (SCH). It asks young people to judge for each item how true it is of them from never (0) to often (2). The possible score range is from 0 to 82 and evidence suggests that scores equal to or greater than 33 effectively discriminate between anxious and non-anxious youth [6].

#### The Strengths and Difficulties of the Questionnaire (SDQ; [23])

We used the parent version SDQ questionnaire (for ages 4–16 years; [24]). This measure includes 25 items across 5 subscales (5 items in each) linked to emotional symptoms, conduct problems, hyperactivity/inattention, peer problems, and pro-social behavior. Items are measured on a 3 point Likert scale reflecting how true the parent feels the statement is for their child (where 0 = not true, 1 = to some extent is true, and 2 = definitely true; score range = 0–10). The SDQ generates a total behavior difficulty score (from 0 to 40) based on items from all subscales (except the prosocial scale which is scored separately). The SDQ shows good reliability and validity [24]. It has been translated into 74 languages and validated in Arabic [3, 4].

The English versions of the SCARED and the SDQ were adapted and translated using the back-translation method. The two scales were translated into Arabic and then back-translated to English. The content of the final translated versions were checked to make sure that they were similar to the original English versions.

## Results

### Approach to Data Analysis

#### Reliability, Validity and Factor Structure

We assessed the internal consistency of the SCARED total and subscale scores using Cronbach’s alpha coefficients. Multivariate analyses of variance (MANOVA) and effect size statistics were using to evaluate gender and age differences for total anxiety and for each subscale. Test–retest reliability was assessed using correlation coefficients. The convergent validity of the SCARED subscale scores was evaluated using correlations between SCARED and SDQ total score and subscale scores. We used CFA to verify whether the five factor structure of the original SCARED questionnaire was a good fit to the population of children and adolescents in Saudi Arabia using the structural equation modeling program AMOS [10].

### Descriptive Statistics

We compared means for the total SCARED and subscale scores between age group (9–12 vs. 13–15-year-olds) and gender. For total anxiety score there was a main effect of age [*F*(1, 938) = 8.40, *p* < .01, *η*^*2*^ = .01] and gender [*F*(1, 938) = 12.41, *p* < .01, *η*^*2*^ = .013]; with children (versus adolescents) and females (versus males) reporting more symptoms. In addition there was a significant interaction between age and gender [*F*(1, 938) = 4.69, *p* < .05, *η*^*2*^ = .005], highlighting that, while adolescent females reported higher levels of symptoms compared with males, there was no gender difference in total anxiety scores for the younger age group. In addition, anxiety symptoms were significantly higher in childhood compared with adolescence for boys and there was no difference between age for females; see Table 1). The analysis for separation anxiety also showed similar gender differences in adolescence and reduced symptoms with age for males. The analysis for social anxiety and school avoidant symptoms showed a main effect of age, highlighting symptom reduction from childhood to adolescence in both subscales. Panic symptoms were higher for females and this was most evident in adolescence (for all significant effects, *F*s > 4 and *p*s < .05). There were no age or gender main effects (and no interaction) for generalized anxiety symptoms (all *F*s < 3 and *p*s > .1).

**Table 1:** Mean total anxiety score and subscale scores including panic (PN), generalized anxiety (GD), separation anxiety (SP), social phobia (SC) and school avoidance (SH) (±SD) for the Screen for Children Anxiety Related Emotional Disorders (SCARED) in children (N = 609; 239 boys) and adolescents (N = 330; 138 boys) and between gender (377 boys; 562 girls)

Subscale	Children		Adolescents		Total by age and gender				Total
	Boys	Girls	Boys	Girls	Children	Adolescents	Boys	Girls	
Total	33.14 ± 12.55	33.88 ± 12.61	28.81 ± 11.82	33.64 ± 11.65	33.51 ± 12.59	31.22 ± 11.93	30.98 ± 12.46	33.70 ± 12.29	32.89 ± 11.46
PN	9.42 ± 4.98	9.52 ± 5.46	8.20 ± 4.64	9.69 ± 4.92	9.48 ± 5.28	9.07 ± 4.85	8.98 ± 4.89	9.581 ± 5.28	9.14 ± 4.76
GD	7.42 ± 3.51	7.49 ± 3.56	6.65 ± 3.51	7.39 ± 3.38	7.46 ± 3.54	7.08 ± 3.45	7.15 ± 3.52	7.46 ± 3.49	7.22 ± 3.93
SP	8.08 ± 3.29	8.86 ± 3.21	6.59 ± 3.12	8.52 ± 3.18	8.56 ± 3.26	7.71 ± 3.29	7.54 ± 3.31	8.75 ± 3.20	8.16 ± 3.23
SC	6.18 ± 3.42	6.32 ± 2.89	5.49 ± 2.58	5.97 ± 2.89	6.26 ± 3.11	5.77 ± 2.77	5.93 ± 3.15	6.12 ± 2.89	6.02 ± 2.90
SH	2.49 ± 1.99	2.49 ± 1.94	2.17 ± 1.75	2.28 ± 1.67	2.49 ± 1.96	2.24 ± 1.69	2.38 ± 1.91	2.42 ± 1.85	2.36 ± 1.77

Correlation analysis showed that each subscale score was correlated with total anxiety (*r*s > .50 and *p*s < .001) and all subscale scores were correlated with each other *r*s > .14 and *p*s < .001). Considering questionnaire cut-offs across the sample, the results showed that 50 % of participants reported elevated anxiety symptoms. For children (males and females) the percentages were 55 % and 52 % and for adolescents they were 38 % and 54 % (males and females).

### Reliability

#### Internal Consistency

The internal consistency was evaluated for children, adolescents and for males and females by calculating Cronbachs alpha coefficients, using a cut-off of >.6 [22]. For total anxiety scores alphas were excellent (all >.80) for each age group and between gender. For subscale scores, alphas across the sample were acceptable (all >.6). Considering subscale scores for child and adolescent, male and female participant groups, these were >.6 for somatic/panic, generalized, separation and social phobia subscales (for males the separation anxiety subscale = .57). In addition, for school avoidance reliability was between .47 and .57 between different groups and .54 overall, indicating poor reliability for this subscale in this population.

#### Test–Retest Reliability

Considering the test–re-test for clinical measures scores ranging from .6 to .8 over a 2 week period are considered satisfactory to excellent, those <.6 fair and <.4 poor [12, 35]. The correlation coefficients between Time 1 and Time 2 for the total SCARED score were all >.7 for the total sample, between gender and for each age group. Correlation coefficients for all other total subscales were all >.5 and no subscale score for either age group or gender fell below 0.50; indicating that stability of this measure in this population was fair to excellent.

### Validity

#### Convergent Validity

The convergent validity of the SCARED was assessed by considering correlations between SCARED total anxiety and subscale scores with the parent report SDQ behavioural difficulties and subscales for a subgroup of participants (n = 267). It was anticipated that SCARED scores would be most clearly associated with the SDQ emotional subscale score. In addition, previous research has found challenges with peer relationships in anxiety, we therefore expected to see a positive association with the SCARED total score and the SDQ peer difficulties subscale. The results showed that while the SDQ emotional and peer difficulties subscales were positively associated with the SCARED total score (respective *r*s = .52 and .38, *p*s < .001), the subscales reflecting conduct and hyperactive symptoms showed a similar relationship (both *r*s = .56, *p*s < .001). The results indicate that parent reported increased behavioural difficulties (for total SDQ and each subscale) were positively associated with child and adolescent self-report anxiety symptoms.

### Confirmatory Factor Analysis (CFA)

We used CFA to test the hypothesized five factor structure proposed in the SCARED. We used several indices to establish the model fit of the five factor hypothesized model, including absolute and incremental or relative fit indices (see [10, 27]). Absolute fit indices are used to test whether the proposed factor model is represented in the current data and they include: Goodness of Fit Index (GFI; range = 0–1, values >.9 indicate a good fit) and Root Mean Square Error of Approximation (RMSEA; range = 0–1, values <.05 indicate a good fit). In addition, we also considered CMIN (the maximum likelihood estimation Chi square and typically expressed as χ^2^); where lower values indicate a better fit and a significant χ^2^ value reflects a poor fitting model). In order to allow some consideration of degrees of freedom in the model to obtain a more pragmatic fit, χ^2^ can also be expressed as a degrees of freedom ratio (χ^2^/*df*), where a value of 1 would indicate a perfect fitting model and values <5 are acceptable and <3 reflect a good fit. Incremental fit indices (to compare the hypothesized factor structure/model to a null model) include the incremental fit index (IFI and the Tucker Lewis index; values are 0–1 and good fit is represented by values >.9) and the comparative fit index that adjust for sample size (CFI; values = 0–1 and >.9 indicate good fit; see [10]).

**Fig. 1:**
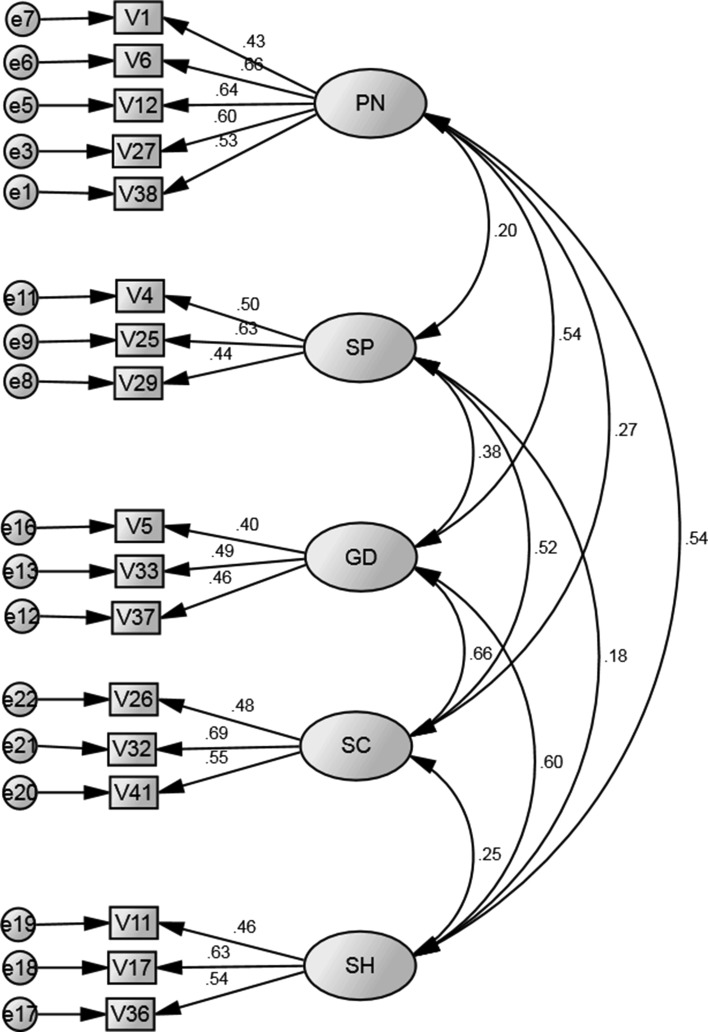
Generated five factor model for school anxiety (SH), panic/somatic symptoms (PN), social anxiety (SC), separation anxiety (SP) and generalized anxiety (GD) (model fit indices are shown in Table 2)

A CFA was run for five factors with each of the 41 subscale items. Factor loadings for this hypothesised model are shown in the “Appendix” and Table 2 outlines all fit indices and shows that this model was not a good fit to the data. To improve model fit, factor loadings less than .4 were removed from the model (following Matsunaga 2010). In addition, model fit was further improved by considering error covariances within each factor and standardised residual covariances for every item in the model. This analysis led to a removal of 17/41 items in the measure and with 4 items in four subscales (GD, SC, SP and SH) and 5 items in one subscale (PN) remaining (see Fig. 1). Table 2 shows the fit indices for the generated model and it highlights that GFI, CFI, IFI and TLI were all >.9 reflecting good model fit. Similarly, RMSEA < .05; indicating good fit. In addition the χ^2^/*df* fit index <3, indicating good fit.

**Table 2:** Generated model and hypothesized five factor model results from the Screen for Anxiety Related Disorders (SCARED) (see Fig. 1)

Model	χ^2^	DF	χ^2^/*df*	GFI	RMSEA	PCLOSE	CFI	TLI	IFI
Hypothesised	2596.01*	796	3.38	.87	.05	.40	.69	.67	.69
Generated	236.48*	109	2.20	.97	.035	1.00	.94	.93	.94

Fit indices included: goodness of fit index (GFI), root mean square error of approximation (RMSEA), the maximum likelihood estimation Chi square (χ^2^ and χ^2^/*df*); the incremental fit index (IFI), the Tucker Lewis index (TLI) and the comparative fit index (CFI)

* *p* < . 000

## Discussion

The current study examined the psychometric properties and factor structure of the Arabic version of the SCARED (total anxiety and subscales reflecting symptoms of panic (PN), generalised anxiety (GD), separation anxiety (SA), social phobia (SC) and school avoidance (SH) in a non-clinical large school sample of children and adolescents in Saudi Arabia. The study explored the pattern of anxiety symptoms between age and gender, and it considered the proportion of young people who reported anxiety symptoms that were greater than the indicated clinical cut-offs. The study also measured the reliability and validity of this screening questionnaire. In addition, it looked at whether the proposed construct measurements (i.e., the five factor model of anxiety) represented a good model fit for this population. The study highlighted some important differences in SCARED scores and factor structure in this Arabic population compared to previous research.

The results indicate that the SCARED is a useful tool to measure anxiety symptoms in an Arabic population. Consideration of the psychometric properties showed that the scale had moderate to good internal consistency for total anxiety and four of the five subscales. In addition test–retest correlations were moderate or good for total anxiety and all subscales. The study therefore supported previous findings of the internal consistency and moderate test–retest reliability in the SCARED [6, 7, 9]. The current study also considered associations between child self-report of anxiety symptoms and parent report of behavioural symptoms more broadly in a subset of the sample. The findings showed positive associations between these two measurements and provided good evidence for convergent validity of the SCARED total score and its five subscales in this population. The results fit well with previous research which has considered links between the SCARED and the SDQ (e.g. [6, 7, 31, 36]).

The study further aimed to examine the suitability of the hypothesized structural model by considering the goodness of fit of the current data to the proposed factor structure reflecting five subscale measurements of panic/somatic symptoms, generalized and separation anxiety, as well as social phobia and school avoidance. The original hypothesized model did not show a good fit to the data. However, the systematic elimination of questionnaire items did generate a five factor model that showed a good fit to the data, indicating that the questionnaire and its subscale measurements could be applied to this population. These findings are consistent with previous studies that supported the SCARED five factor structure [16, 28, 39, 43]. The reduction of items within each scale indicates that a narrower set of items reflects anxiety in children and adolescents in Saudi Arabia (e.g., symptoms associated with shyness for social anxiety or being alone or away from home/family for separation anxiety). Further research should aim to replicate this result in a further sample of children and adolescents in this country and Arabic cultures more widely.

Considering patterns of age and gender, the study is consistent with most studies (e.g. [13–15, 28]) demonstrating elevated anxiety symptoms in girls compared to boys (where this difference was evident for total anxiety symptoms and subscales measuring panic and separation anxiety). In addition, the data showed reporting of anxiety symptoms diminished with age for total anxiety and each subscale, and where this reduction was significant for all variables except generalized anxiety disorder) and only evident for boys with respect to total anxiety, panic and separation symptoms (with girls showing stable symptoms over time). The results support previous research showing that in adolescence girls report increased emotional symptoms (including anxiety and depression) compared with boys (e.g. [42]). One notable feature of the current data was that gender interacted with age, reflecting reduced total anxiety symptoms in boys from childhood to adolescence and a stable pattern of (comparatively) elevated symptoms across time in girls. The results highlight that girls in Saudi Arabia are at increased risk of experiencing elevated and chronic anxiety symptoms. Further longitudinal research is needed to explore these developmental and gender differences and specifically the different pathways between gender (i.e., the stable symptom pathway in girls and the high decreasing pattern seen in boys in this sample).

Considering clinical cut-offs on this measurement, the results found that a large proportion of children and adolescents (<50 %) endorsed anxiety symptoms and met the clinical cutoff for the SCARED. The results showed that around half of children (males and females) showed elevated symptoms using this screen, and that this percentage was also true for female adolescents (scores for adolescent males were typically reduced relative to the child scores). Other studies have found that this screening measure has identified around one-third of African American adolescents showing elevated symptoms [9] and similar scores were found in a population of children from South Africa [37, 39]. In addition, subscale scores in this sample were largely equivalent to other samples including Dutch children and adolescents [28], but were greater than a Chinese school sample of adolescents [43]. It is possible that cultural differences might account for elevated scores in children and adolescents in Saudi Arabia and further research should aim to understand the elevated endorsement of symptoms in this population. One alternative explanation of these results is that the use of a three point Likert scale can potentially lead to an over reporting of anxiety symptoms in some populations. For example, Krosnick and Presser [34] noted that “moderate attitudes” (p. 269; those that do not sit in the neutral midpoint of a scale) can be difficult to endorse on a three point scale. They suggest that the inclusion of a Likert scale with five response options allows for a response that reflects some endorsement of a symptom (e.g., little or somewhat) and that does not require responding to either extreme point of a response scale. Modification of the response scale could potentially lead to a different score profile for children and adolescents in Saudi Arabia.

## Summary

This study explored anxiety symptoms in a population of children and adolescents in Saudi Arabia using the SCARED. The results showed that this questionnaire was a reliable and valid tool for use in this population. In addition, the five factor structure represented a good fit to the data. The emergence of anxiety in this population and the different symptom profile for girls and boys (though somewhat elevated) was similar to the pattern of anxiety symptoms in other countries. They highlight that while boys showed a reduction of anxiety symptoms with age, girls showed stable and elevated anxiety symptoms over time, suggesting that they would benefit from a universal intervention at an early age to address the development of further mental health difficulties and adverse developmental outcome. The results are important for understanding cross cultural differences in disorders that can emerge early in childhood and that have a significant negative impact on development.

## Appendix

**Table:** Confirmatory factor analysis for the five subscales for the Screen for Anxiety Related Disorders (SCARED) highlighting initial factor loadings for the hypothesized model

Code	Attributes	PN	GD	SP	SC	SH
**V1**	**When I feel frightened, it’** **s hard to breathe**	.44				
**V6**	**When I get frightened, I feel like passing out**	.58				
V9	People tell me that I look nervous	.16				
**V12**	**When I get frightened, I feel like I am going crazy**	.58				
V15	When I get frightened, I feel like things are not real	.38				
V18	When I get frightened my heart beats fast	.22				
V19	I get shaky	.43				
V22	When I get frightened, I sweat a lot	.31				
V24	I get really frightened for no reason at all	.44				
**V27**	**When I get frightened, I feel like I am choking**	.56				
V30	I am afraid of having anxiety (or panic) attacks	.33				
V34	When I get frightened, I feel like throwing up	.46				
**V38**	**When I get frightened, I feel dizzy**	.52				
V14	I worry about being as good as other kids		.46			
**V5**	**I worry about other people liking me**		.41			
V7	I am nervous		.22			
V21	I worry about things working out for me		.36			
V23	I am a worrier		.58			
V28	People tell me that I worry too much		.44			
**V33**	**I worry about what is going to happen in the future**		.41			
V35	I worry about how well I do things		.34			
**V37**	**I worry about things that have already happened**		.44			
**V4**	**I get scared if I sleep away from home**			.49		
V8	I follow my mother or father wherever they go			.34		
V13	I worry about sleeping alone			.51		
V16	I have nightmares about something bad happening to my parents			.21		
V20	I have nightmares about something bad happening to me			.28		
**V25**	**I am afraid to be alone in the house**			.61		
**V29**	**I don’** **t like to be away from my family**			.41		
V31	I worry that something bad might happen to my parents			.37		
V3	I don’t like to be with people I don’t know well				.32	
V10	I feel nervous with people I don’t know well				.20	
**V26**	**It is hard for me to talk with people I don’** **t know well**				.51	
**V32**	**I feel shy with people I don’** **t know well**				.60	
V39	I feel nervous when I am with other children or adults				.40	
V40	I feel nervous when I am going to parties, dances, or…				.30	
**V41**	**I am shy**				.53	
V2	I get headaches when I am at school					.29
**V11**	**I get stomach aches at school**					.47
**V17**	**I worry about going to school**					.60
**V36**	**I am scared to go to school**					.56

Bolded items were retained in the final generated five factor model

*PN* panic/somatic symptoms, *SP* separation anxiety, *SC* social anxiety, *GD* generalized anxiety, *SH* school avoidance
